# Supplementary figures and images for: The Population Dynamics and Parasitism Rates of Ceratitis capitata, Anastrepha fraterculus, and Drosophila suzukii in Non-Crop Hosts: Implications for the Management of Pest Fruit Flies
Source: Insects. 2024 Jan 15;15(1):61. doi: 10.3390/insects15010061 (PMC10817041; doi:10.3390/insects15010061)

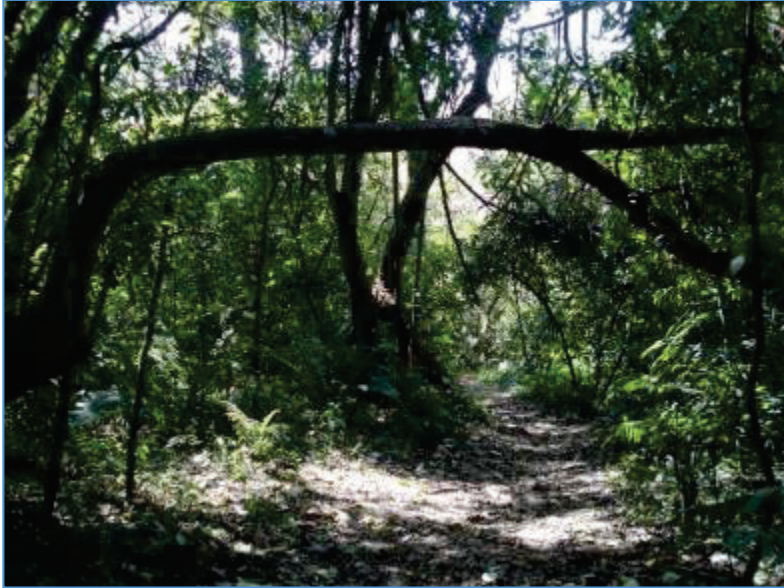

Supplement: Supplementary file 1 [file insects-15-00061-s001.zip › insects-2801764-File S1.pdf]

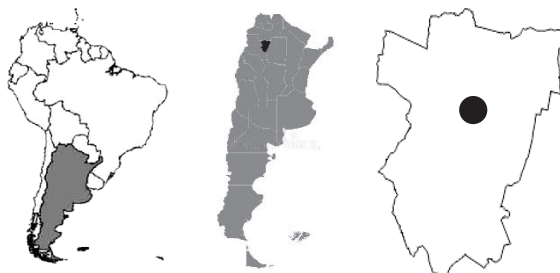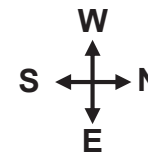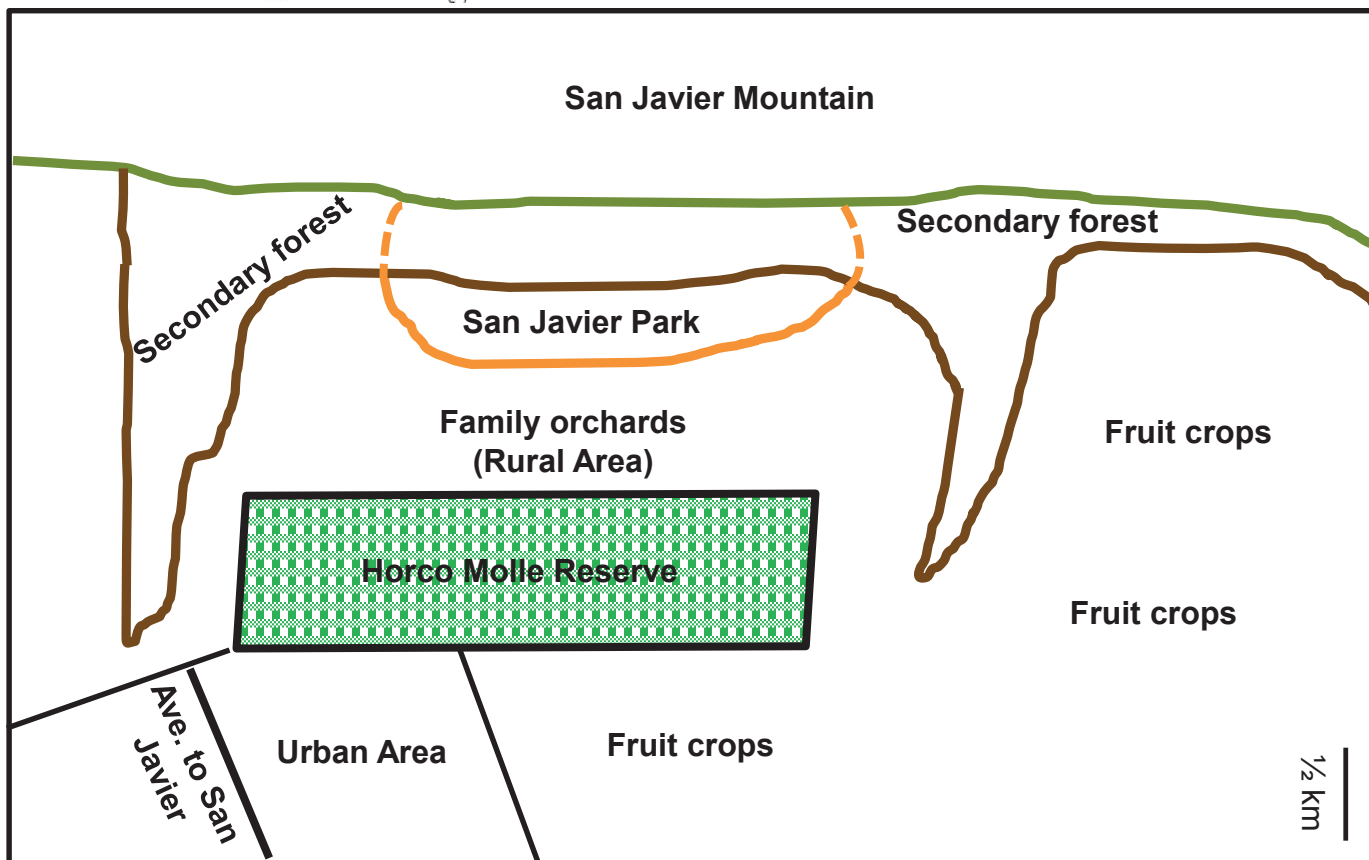

Supplement: Supplementary file 1 [file insects-15-00061-s001.zip › insects-2801764-File S2.pdf]
